# Macrophages and pulmonary fibrosis: a bibliometric and visual analysis of publications from 1990 to 2023

**DOI:** 10.3389/fmed.2024.1374177

**Published:** 2024-06-17

**Authors:** Yi Min, Lifei Wu, Cheng Xu, Wen Han, Zhi Yin, Xu Pan, Luyao Sun, Jinrong Zhang, Guoqiang Wan, Tongxin Zhou

**Affiliations:** ^1^Yixing Traditional Chinese Medicine Hospital, Wuxi, Jiangsu, China; ^2^Key Laboratory of Biomarkers and In Vitro Diagnosis Translation of Zhejiang Province, Hangzhou, Zhejiang, China; ^3^Department of Physical Education, Zhejiang Chinese Medical University, Hangzhou, Zhejiang, China; ^4^Fifth People’s Hospital of Suzhou, Suzhou, Jiangsu, China

**Keywords:** macrophages, pulmonary fibrosis, bibliometric, VOSviewer, CiteSpace, KEGG

## Abstract

**Background:**

The role of macrophages in the symptomatic and structural progression of pulmonary fibrosis (PF) has garnered significant scholarly attention in recent years. This study employs a bibliometric approach to examine the present research status and areas of focus regarding the correlation between macrophages and PF, aiming to provide a comprehensive understanding of their relationship.

**Methodology:**

The present study employed VOSviewer, CiteSpace, and Microsoft Excel software to visualize and analyze various aspects such as countries, institutions, authors, journals, co-cited literature, keywords, related genes, and diseases. These analyses were conducted using the Web of Science core collection database.

**Results:**

A comprehensive collection of 3,479 records pertaining to macrophages and PF from the period of 1990 to 2023 was obtained. Over the years, there has been a consistent increase in research literature on this topic. Notably, the United States and China exhibited the highest level of collaboration in this field. Through careful analysis, the institutions, authors, and prominent journals that hold significant influence within this particular field have been identified as having the highest publication output. The pertinent research primarily concentrates on the domains of Biology and Medicine. The prevailing keywords encompass pulmonary fibrosis, acute lung injury, idiopathic pulmonary fibrosis, and others. Notably, TGFβ1, TNF, and CXCL8 emerge as the most frequently studied targets, primarily associated with signaling pathways such as cytokine–cytokine receptor interaction. Additionally, cluster analysis of related diseases reveals their interconnectedness with ailments such as cancer.

**Conclusion:**

The present study employed bibliometric methods to investigate the knowledge structure and developmental trends in the realm of macrophage and PF research. The findings shed light on the introduction and research hotspots that facilitate a more comprehensive understanding of macrophages and PF.

## Introduction

Pulmonary fibrosis (PF) is a destructive lung disease caused by various etiologies, representing the result of the body’s self-repair mechanisms following lung injury. Among its hallmark features are damage to alveolar epithelial cells, proliferation of fibroblasts, and excessive deposition of extracellular matrix proteins. The progressive process ultimately results in respiratory failure and death (1). The data indicate that PF affects approximately three million people globally (2). Furthermore, as the world’s population continues to age, the incidence of PF is also increasing (3).

Currently, there are limited treatment options for PF. Commonly used anti-PF medications include nintedanib and pirfenidone. While these drugs can slow the decline in lung function and prolong survival, their use is associated with significant toxic side effects, potentially leading to severe complications (4). To date, lung transplantation remains the only potential cure for end-stage PF patients. However, despite this option, limitations exist due to the risk of allograft failure caused by acute and chronic rejection reactions. The median survival rate at 6–7 years post lung transplantation is among the lower rates observed for solid organ transplants (5).Therefore, there is a pressing need to delve into the physiopathological mechanisms of PF and actively explore novel therapeutic strategies.

There are two types of macrophages in the lungs: alveolar macrophages (AMs) and interstitial macrophages (IMs). These macrophages are crucial in maintaining the homeostasis and facilitating tissue remodeling in PF (6). Persistent alveolar injury and incomplete repair play pivotal roles in the pathogenesis of PF. This incurable condition is further aggravated by aberrant lung repair following epithelial injury, which is closely associated with macrophage infiltration (7). Consequently, macrophages are implicated in the pathogenesis of PF, and investigating the interplay between macrophages and PF holds promise for advancing therapeutic interventions.

In recent times, there has been extensive utilization of bibliometric analysis to examine extensive scientific research data and discern emerging trends (8). Crucially, it has the capability to encapsulate the evolution of publications, forecast research hotspots, and assess frontiers within specific fields through the use of a citation network (9–11). In spite of the fact that relevant academic researchers have published bibliometric analyses on PF, no similar analyses have yet been published about macrophages in PF (12). Significantly, various bibliometric tools, including CiteSpace, VOSviewer, and the R package “bibliometrix,” have been utilized to visually represent specific fields of medical literature analysis (13). Hence, this current study employed bibliometric analysis to address this knowledge gap. The paper conducted a comprehensive examination of literature pertaining to macrophages associated with PF and performed visualization analysis spanning the last three decades (from 1990 to 2023). The aim was to identify significant features and anticipate future research directions in this domain.

## Materials and methods

### Data sources and retrieval strategies

**FIGURE 1:**
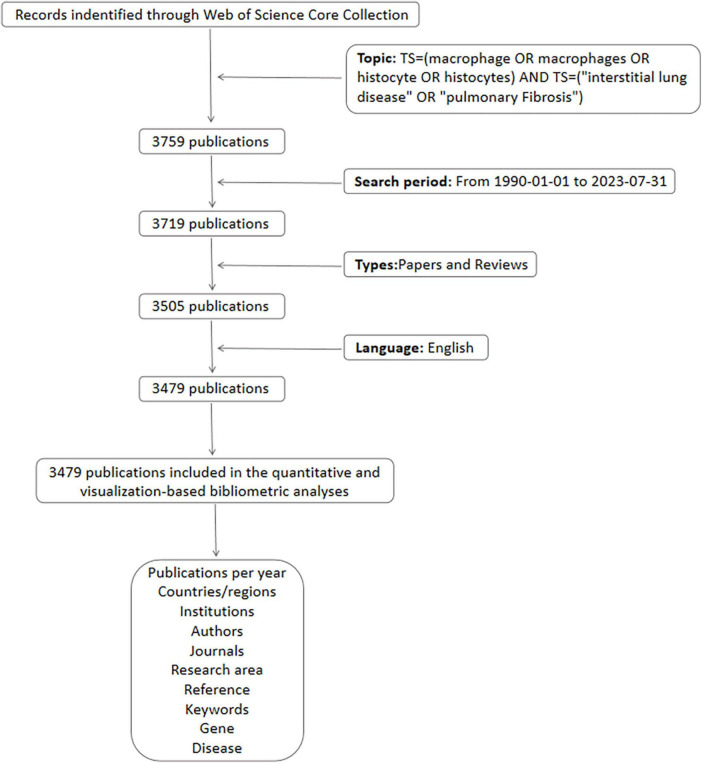
Flowchart of literature collection and selection.

The Web of Science core collection database was accessed through the WOS database using the search method TS = (macrophage OR macrophages OR histiocyte OR histiocytes) AND TS = (“internal lung disease” OR “common fiber”), with a specified search time frame from January 1990 to 31 July 2023. The inclusion criteria encompassed papers and reviews relevant to retrieval, while excluding letters, briefs, book reviews, and similar content. This search yielded a total of 3,479 articles, which were subsequently utilized for visual analysis pertaining to countries, institutions, authors, journals, co-cited literature, and keywords (Figure 1).

### Citexs data analysis platform

Gene and disease data are sourced from the Citexs platform^1^ for visualized analysis of genes and diseases.

### Research methods

A bibliometric approach was employed to analyze the retrieved literature using visualization tools such as VOSviewer 1.6.18 (Centre for Science and Technology Studies, Leiden University, The Netherlands), CiteSpace 6.1.6 (Chaomei Chen, China), Pajek 64 5.16 (University of Ljubljana, Slovenia), Scimago Graphica 1.0.35^2^ (USA), and Microsoft Excel (Microsoft Office 2021, Microsoft, Redmond, WA, USA). Nationalities, institutions, authors, journals, co-cited references, keywords, genes, and diseases were subjected to visual analysis. The information on genes and diseases was sourced from the Citexs platform (see text footnote 1). Visualization maps were generated to analyze the current status, hotspots, and trends of this research.

Utilizing the R language packages Clusterprofiler, enrichplot, and ggplot2, we performed visualizations for Gene Ontology (GO) enrichment analysis and Kyoto Encyclopedia of Genes and Genomes (KEGG) enrichment analysis on the extracted genes. For the construction analysis and visualization of Protein–Protein Interaction (PPI) networks, we employed the STRING online platform^3^ and Cytoscape 3.8.2 (Cytoscape Consortium, USA).

The trend of keyword heatmaps over time was analyzed using Scimago Graphica 1.0.35 (see text footnote 2, USA).

VOSviewer 1.6.18 and Pajek 64 5.16 (University of Ljubljana, Slovenia) were utilized for co-occurrence analysis of countries, institutions, authors, journal publications, research fields, frequency of hotspot terms, genes, and diseases. Different colors in the analyses of countries, institutions, authors, research fields, frequency of hotspot terms, genes, and diseases represent different clusters. In the generated graphs, each node is represented by a sphere and a text label, with the size of the sphere indicating the node’s size. Lines between nodes represent co-occurrence relationships, and the thickness of connecting lines indicates the strength of co-occurrence. In the analysis of journal publications, the area and color depth of red patches are directly proportional to the quantity of publications.

## Results

### Annual publication trend analysis

**FIGURE 2:**
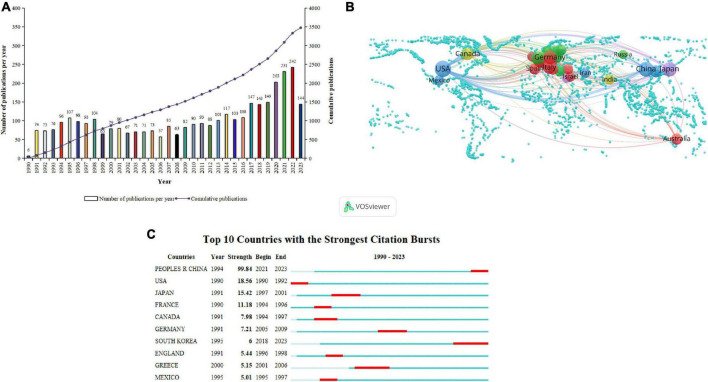
**(A)** Analysis of publication trends. **(B)** Co-occurrence analysis of research regions. **(C)** Current picture of the study area.

From 1 January 1990, to 31 July 2023, the cumulative number of publications related to macrophages and PF was 3,479, with an average annual publication rate of 102.32 papers. As depicted in Figure 2A, there are two peaks in publications during this period, in 1995 and 2022. This indicates that research outcomes related to the association between macrophages and PF were particularly prolific in these 2 years, with a concentrated output, signifying periods of heightened research focus. Figure 2A illustrates an overall increasing trend in total publications over the years, suggesting a rising research interest in macrophage-related PF, which holds research significance. Complete data can be found in Supplementary Table 1.

### Analysis of regional relationships in research

Figure 2B illustrates a visual analysis of the publication regions using VOSviewer software. A total of 69 countries have contributed to 3,479 articles related to macrophages and PF. The map includes regions with a minimum publication count of 10 articles, forming a collaborative network in the research on macrophages and PF. Each circle represents a country, and the thickness of the connecting lines indicates the strength of collaboration between countries. The size of each circle is proportional to the number of publications from that country.

From Figure 2B, it is evident that the United States demonstrates the strongest collaboration inclination with other countries. The collaboration between the United States and China appears to be particularly close. The United States has the highest publication count, contributing 1,297 articles, which constitutes 31.47% of the total publications. Following closely are China and Japan with publication counts of 601 and 471 articles, respectively. Complete data can be found in Supplementary Table 2.

Figure 2C presents an analysis conducted through CiteSpace on the top 10 countries with a surge in publications related to macrophages and PF from 1 January 1990, to 31 July 2023. The burst detection map identifies periods of a sharp increase in publication output for each country, with the red areas indicating the specific time frames of increased publication activity.

From Figure 2C, it is observed that during this period, China exhibited the highest burst strength, with a burst strength value of 99.84, and the burst period spanning from 2021 to 2023. Among the countries analyzed, South Korea and Greece had the longest burst periods, spanning from 2018 to 2023 and 2001 to 2006, respectively. This suggests that these two countries have shown a pronounced emphasis on research related to the correlation between macrophages and PF during the respective time periods.

### Relationship analysis of research institutions

**FIGURE 3:**
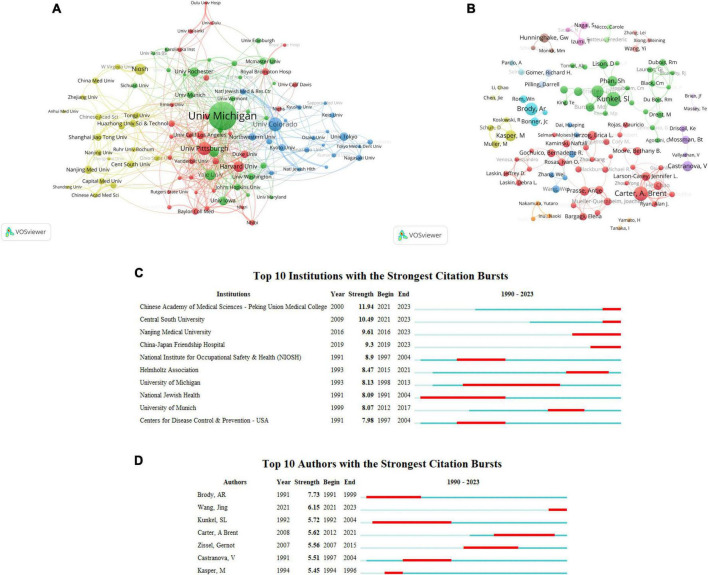
**(A)** The relationship analysis diagram of the research institutions. **(B)** Institutional publication surge analysis. **(C)** Co-authorship analysis of research authors. **(D)** Visualization of research authors’ publication emphasis.

Through VOSviewer software, a visualization analysis was conducted on research publication institutions. A total of 2,967 institutions have contributed to the 3,479 publications on the correlation between macrophages and PF. By setting the minimum publication threshold for institutions to 13 articles, the top 100 collaborating institutions in research on the correlation between macrophages and PF were identified, as shown in Figure 3A. Different colors represent distinct clusters based on the co-citation network between institutions, where institutions with high co-citation relationships are grouped together, forming a hierarchical clustering structure to illustrate the relationships between institutions. The thickness of the lines connecting circles reflects the strength of collaboration between institutions, and the size of the circles is proportional to the number of publications from each institution.

Figure 3A reveals that Harvard University demonstrates the strongest willingness to collaborate with other institutions. The collaboration between the University of Colorado and the National Jewish Medical and Research Center is particularly close. The University of Michigan has the highest publication output, with 107 articles, ranking first. Following closely are the University of Colorado and the University of Pittsburgh, with 52 and 50 articles, respectively. Complete data can be found in Supplementary Table 3.

Figure 3B presents the analysis conducted by CiteSpace on the top 10 institutions with a surge in research publications on the correlation between macrophages and PF from 1 January 1990, to 31 July 2023. The institution surging graph illustrates a significant increase in publication output for each institution during specific time intervals, represented by the red-colored regions. According to Figure 3B, during the period from 2021 to 2023, the Chinese Academy of Medical Sciences – Peking Union Medical College experienced a surge in research publications. Over the past 3 years, institutions that have demonstrated a notable increase in publication output include the Chinese Academy of Medical Sciences – Peking Union Medical College, Central South University, Nanjing Medical University, and China-Japan Friendship Hospital. This suggests that these institutions have recently placed greater emphasis on research related to the correlation between macrophages and PF. Complete data can be found in Supplementary Table 4.

### Relational analysis of the study authors

Through visual analysis using VOSviewer software, a comprehensive examination of research publishing entities was conducted. A total of 17,700 authors contributed to 3,479 research articles related to the association between macrophages and PF. A threshold of a minimum of eight publications per author was set to construct a collaborative network graph among researchers focused on macrophage-PF relationships. As illustrated in Figure 3C, each node in the graph is composed of a circle and a corresponding text label, representing a researcher. Different colors denote distinct clusters.

Brent Carter exhibited the highest inclination for collaboration among all authors. Line thickness represents the strength of collaboration between authors, with A. Brent Carter demonstrating the closest collaboration with Jennifer L. Larson-Casey. Additionally, the size of each circle is positively correlated with the number of research articles authored by the respective individual. A. Brent Carter and Sl Kunkel lead the publication count with 25 articles each, securing the top position. Following closely are Ar Brody and Rm Strieter, with publication counts of 22 and 21 articles, respectively.

Figure 3D illustrates the emergence of the top 10 authors in terms of research publications on the correlation between macrophages and PF from 1 January 1990, to 31 July 2023, as analyzed using CiteSpace. The Author Emergence Chart depicts a sudden surge in publication activity for individual authors during specific time intervals, represented by the red-shaded areas in the graph. According to Figure 3D, Brody, AR experienced a significant increase in publication output from 1991 to 1999, with the initial publication occurring in 1991. Over the past 3 years, Wang, Jing stands out as an author with a notable surge in publication output, indicating a recent emphasis on research related to the correlation between macrophages and PF.

### Journal analysis

**FIGURE 4:**
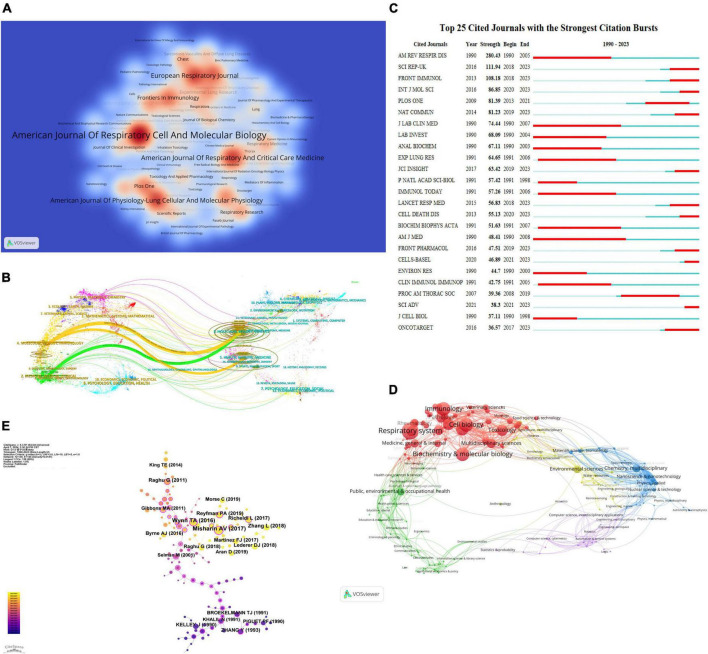
**(A)** Thermal map of journal publication. **(B)** Journal double superposition graph. **(C)** Journal citation emergence chart. **(D)** Field analysis chart. **(E)** Co-citation analysis of literature.

Through visual analysis using VOSviewer software, a total of 827 journals collectively published 3,479 research articles related to the correlation between macrophages and PF. To generate a heat map of journal publications focusing on macrophage-PF relationships, a minimum publication threshold of seven articles per journal was set. The color intensity in the heat map corresponds to the publication volume, with darker shades indicating higher publication numbers. The journal with the highest publication output is the “American Journal of Respiratory Cell and Molecular Biology” contributing 176 articles. Following closely are the “American Journal of Respiratory and Critical Care Medicine” and “American Journal of Physiology-Lung Cellular and Molecular Physiology” with publication counts of 107 and 102 articles, respectively, as shown in Figure 4A. Complete data can be found in Supplementary Table 5.

Figure 4B presents an overlaid dual-map analysis of journals, revealing the positioning of research themes related to macrophages and PF in relation to major disciplinary areas. Right and left sides of the figure represent cited and citing journals, respectively. The analysis indicates that research publications on the correlation between macrophages and PF are predominantly concentrated in journals within the fields of Molecular Biology, Immunology, and Clinical Medicine. On the other hand, the cited journals are concentrated in the fields of Molecular Biology, Genetics, and Health, Nursing, and Medicine.

In the figure, points represent journals and curves indicate citation connections between left and right sections. Insights into the interdisciplinary relationships within the field are revealed by the paths and origins of citations, as depicted comprehensively by the trajectories of these connections.

Figure 4C depicts the top 25 cited journals that emerged in research on the correlation between macrophages and PF from 1 January 1990, to 31 July 2023, as analyzed using CiteSpace. The Journal Emergence Chart illustrates instances where a journal experiences a substantial increase in citation frequency during specific time intervals, represented by the red-shaded areas in the graph. Over the past 3 years, the top three journals with significant emergence and intensity of emergence in the field of macrophage-related PF are SCI REP-UK, FRONT IMMUNO, and INT J MOL SCI. This suggests a notable increase in citation frequency for these journals, indicating a growing recognition and attention within the realm of macrophage-related PF research.

### Research field analysis

A total of 226 literature categories were identified from the search of the Web of Science Core Collection database. Through statistical analysis and visualization using VOSviewer software, 3,479 research articles on the correlation between macrophages and PF were clustered into five major domains. As illustrated in Figure 4D, different-colored spheres represent distinct domain clusters, with the primary focus of macrophage-related PF research concentrated in the field of Biology and Medicine. Within this domain, subfields such as Zoology and Women’s Studies exhibit notable proportions. Complete data can be found in Supplementary Table 6.

### Citation analysis of literature

Utilizing CiteSpace, we conducted a citation analysis of literature on the correlation between macrophages and PF from 1 January 1990, to 31 July 2023. The data presented in the figure is based on the following parameters set in CiteSpace: time slices (1990–2023), each slice representing a 2-year interval, and selection criteria (*k* = 1). The size of overlaid circles on the annual rings corresponds to the cumulative citation count, with shades ranging from magenta (indicating earlier citations) to yellow (indicating later citations). The color overlay signifies that the article was cited in the corresponding year.

Interconnecting lines between circles represent co-citation relationships, and nodes marked in rose red indicate key nodes with centrality greater than 0.1. From Figure 4E, a total of 126 citations have been cited in the article titled “Monocyte-derived alveolar macrophages drive lung fibrosis and persist in the lung over the life span.” Complete data can be found in Supplementary Table 7.

### Cluster analysis of hotspot term frequencies

Using VOSviewer software, co-occurrence cluster analysis was conducted on keywords, setting a minimum occurrence threshold of 8 times per keyword. Out of 4,899 unique keywords (reduced to 4,795 after deduplication and merging), 148 keywords meeting the specified criteria were selected for visualization in the graph.

**FIGURE 5:**
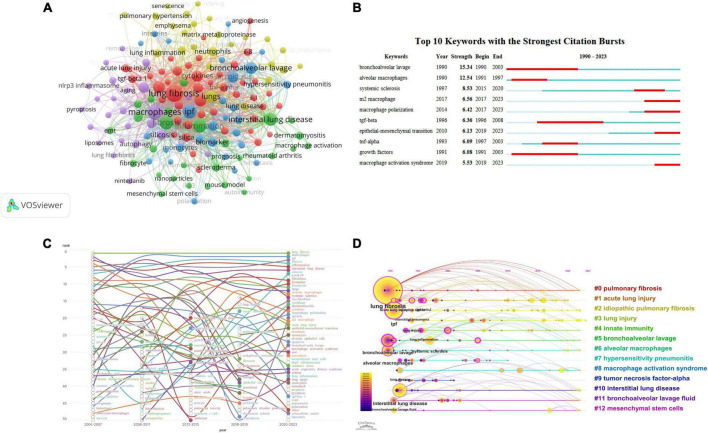
**(A)** Co-occurrence analysis of hotspot keyword frequencies. **(B)** Emergence chart of hotspot keyword frequencies. **(C)** Evolution of keyword popularity over the last two decades. **(D)** Timeline analysis of hotspot keyword co-occurrence clustering.

As depicted in Figure 5A, each node is composed of a circle and a label, with circle size proportional to the frequency of keyword occurrence. Based on a co-occurrence clustering algorithm, different-colored nodes form distinct clusters representing 11 different thematic areas. Similarity between keywords was computed, and highly similar keywords were grouped together. Notably, the red cluster focuses on etiological research, with “lung fibrosis” being the most prominent term. The yellow cluster pertains to disease nomenclature, with “lungs” as the most prominent term. The green cluster is related to pathogenesis research, with “fibrosis” being the most prominent term. The blue cluster is associated with diagnostic research, with “ipf” (idiopathic pulmonary fibrosis) as the most prominent term. Finally, the purple cluster is dedicated to pathological research, with “macrophages” as the most prominent term. Complete data can be found in Supplementary Table 8.

Figure 5B presents the top 10 emerging keywords in research on the correlation between macrophages and PF from 1 January 1990, to 31 July 2023, as analyzed using CiteSpace. The Keyword Emergence Chart illustrates instances where a keyword experiences a substantial increase in frequency during specific time intervals, represented by the red-shaded areas in the graph. Over the past 3 years, the emerging keywords include m2 macrophage, epithelial-mesenchymal transition, and macrophage activation syndrome indicating a recent surge in articles related to these keywords in the field of macrophage-related PF research, potentially signifying new research trends.

### Keyword heatmap analysis

A frequency analysis using articles from 2004 to 2022 was conducted, ranking keywords every 4 years. The top 50 keywords for each time period were plotted to generate a trend graph depicting the keyword popularity over 20 years. The fluctuating curves reflect changes in ranking trends. Hollow circles indicate the first appearance within the top 50 from 2003 to 2022, while solid circles represent the final time period of being within the top 50, as illustrated in Figure 5C. Complete data can be found in Supplementary Table 9.

During the period from 2004 to 2007, the keywords “lung fibrosis,” “macrophages,” “ipf,” and “silicosis” entered the top 50 for the first time. Over time, their rankings exhibited an upward trend, suggesting an increasing attention to PF and macrophages.

Using CiteSpace, we conducted a timeline analysis of the co-occurrence and clustering of hotspot keyword frequencies related to macrophages and PF from 1 January 1990, to 31 July 2023. The sizes of overlaid circles on annual rings were proportional to the cumulative keyword frequencies. Keywords were positioned at the top of the view, with time progressing from left to right. The color spectrum ranged from magenta (earlier appearance) to yellow (later appearance), with the color overlay indicating the keyword’s presence in the corresponding years.

Interconnecting lines between circles represented co-occurrence relationships, and nodes marked in rose red were key nodes with centrality greater than 0.1. Clusters of keywords were arranged horizontally. The number of keywords within each cluster reflected their respective frequencies, with higher frequencies indicating greater popularity. As shown in the figure, the keywords clustered into 13 groups, including “pulmonary fibrosis,” “acute lung injury,” and “idiopathic pulmonary fibrosis,” among others.

In Figure 5D, the keyword clustering module value (Q) is 0.8512, and the average silhouette value (S) is 0.8739. CiteSpace calculates these values based on network structure and clustering clarity. Typically, Q values exceeding 0.3 indicate a significant clustering structure, while S values surpassing 0.5 suggest reasonable clustering. Therefore, in the upper-left region of Figure 5D, it is observed that the clustering module value Q is 0.8512, and the average silhouette value S is 0.8739. Both Q (0.8512 > 0.3) and S (0.8739 > 0.5) exceed the respective threshold values, supporting the conclusion that the clustering structure of the sample keywords is significant and convincing. Complete data can be found in Supplementary Table 10.

### Cluster analysis of associated genes

**FIGURE 6:**
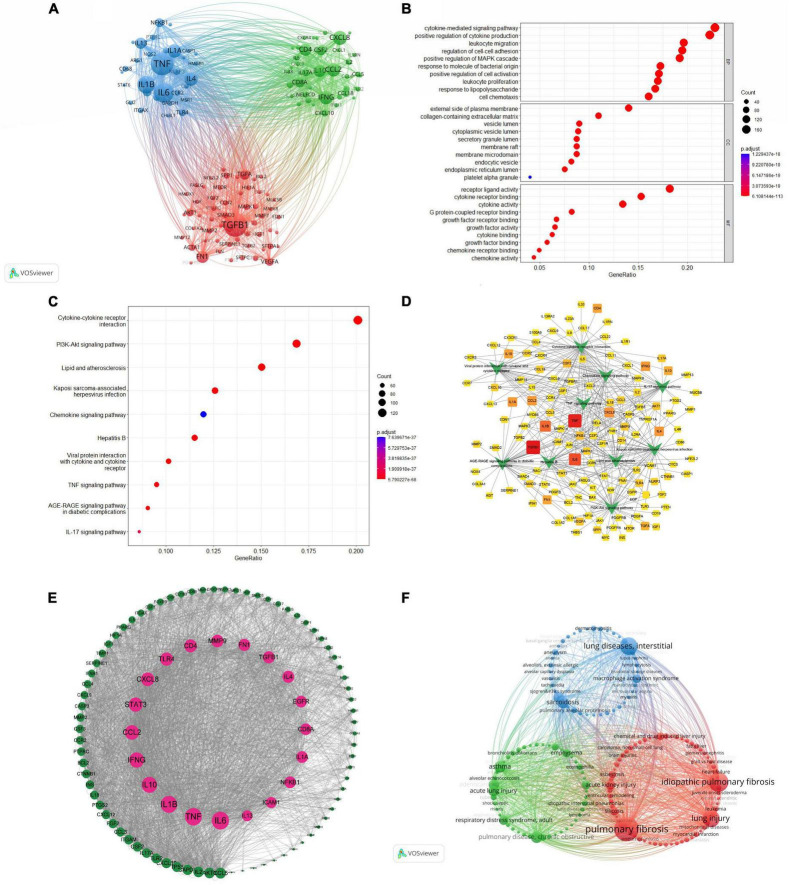
**(A)** Analysis of associated genes. **(B)** Bubble plot of GO enrichment analysis. **(C)** Bar plot of KEGG pathway enrichment analysis. **(D)** The core genes corresponding to the top 10 signaling pathways. **(E)** Analysis of PPI. **(F)** Analysis of associated diseases.

Using VOSviewer software, co-occurrence clustering analysis of genes associated with macrophages and PF research was conducted. The Citexs Big Data platform extracted a total of 3,299 genes from 3,479 articles, setting a minimum occurrence threshold of 30 times for each gene (authors meeting these criteria were included in the visualization) (Figure 6A). The resulting visual map comprises nodes represented by circles and labels, with the size of each circle proportional to the frequency of gene occurrence. The thickness of lines connecting circles reflects the strength of relationships between genes. Different-colored nodes form distinct clusters, representing gene clusters in different domains. Notably, the red cluster is led by TGFβ1, the blue cluster by TNF, and the green cluster by CXCL8, each exhibiting the highest popularity within their respective gene clusters. Complete data can be found in Supplementary Table 11.

### GO and KEGG enrichment analysis

Utilizing the Citexs Big Data platform, a total of 771 genes, each appearing more than 4 times in the articles, underwent GO and KEGG enrichment analysis. The analysis was visualized using the R package Clusterprofiler for comprehensive and informative representation.

The results of GO functional enrichment were presented in Figure 6B, covering Biological Processes (BP), Molecular Functions (MF), and Cellular Components (CC). In Biological Processes, genes were enriched in biological functions such as the “cytokine-mediated signaling pathway.” In Cellular Components, gene enrichment was observed in biological functions like the “external side of the plasma membrane.” Regarding Molecular Functions, gene enrichment was seen in biological functions such as “receptor ligand activity.” Complete data can be found in Supplementary Table 12.

The top 20 significantly enriched pathways were selected for the KEGG pathway enrichment analysis, and a bar plot was generated for each. Genes that are significantly enriched in each pathway are represented by the *X*-axis, while the *Y*-axis represents different signaling pathways. Longer bars indicate a higher number of genes and greater significance after enrichment. As shown in Figure 6C, this study is primarily associated with signaling pathways such as “cytokine–cytokine receptor interaction.”

### The core genes corresponding to the top 10 signaling pathways

Figure 6D depicted the correlation between core genes and signaling pathways. The squares in the graph represented core genes, with darker shades indicating higher frequencies of occurrence. Green arrows denoted the top 10 signaling pathways. The figure elucidated the relationship between macrophages and high-frequency genes and pathways associated with PF. For instance, TGFβ1 was primarily associated with the AGE-RAGE signaling pathway in diabetic complications, and cytokine–cytokine receptor interaction pathways. These interconnected associations comprehensively delineated the relationship between key genes and relevant signaling pathways in PF and its pathogenesis. Complete data can be found in Supplementary Table 13.

### Analysis and construction of PPI network

Utilizing the Citexs Big Data platform, the top 100 proteins based on article occurrence were imported into the STRING platform (minimum protein occurrence set at 38 times) with the species specified as “Homo sapiens.” A PPI network was constructed with high confidence (0.700) settings. The obtained network information was then imported into Cytoscape software to calculate node degree values.

After importing the filtered proteins into the STRING database, a PPI network was generated, consisting of 100 nodes and 1,478 edges, with an average node degree value of 29.6. Using Cytoscape software, node degree values were calculated and a core protein PPI network diagram was constructed by sorting nodes in ascending order based on degree values, as shown in Figure 6E. The top five proteins in terms of degree values are IL6, TNF, IL1B, IL10, and IFNG, indicating that these proteins may serve as core proteins in the network. Complete data can be found in Supplementary Table 14.

### Analysis of association diseases

Citexs Big Data platform extracted a total of 1,762 diseases from 3,479 articles. Through VOSviewer software, co-occurrence clustering analysis was conducted, setting a minimum occurrence threshold of 20 times for each disease (diseases meeting these criteria were included in the visualization) (Figure 6F). Nodes are represented by circles and labels, with circle size proportional to disease frequency. The thickness of lines connecting circles reflects the strength of relationships between diseases. Different-colored nodes form distinct clusters, representing disease clusters in different domains. Notably, the red cluster pertains to the field of “Damaging Diseases,” with PF being the most prominent. The green cluster represents diseases related to tumors, with “Pulmonary Disease,” “Chronic Obstructive” as the most prominent. The blue cluster corresponds to the field of “Inflammatory Diseases,” with “Lung Diseases,” “Interstitial” being the most prominent. Complete data can be found in Supplementary Table 15.

## Discussion

This literature-based quantitative study represents the first retrospective analysis of research trends pertaining to macrophages in the field of PF. Our findings elucidate the current status, trends, and focal points of research related to macrophages and PF. A comprehensive analysis of 3,479 articles reveals a consistent upward trajectory in literature addressing macrophages and PF over the past three decades. Notably, there is a significant surge in publications from 2020 to 2022, indicating a heightened attention to the relationship between macrophages and PF in recent years. This increased focus underscores the evolving nature of research in this domain, suggesting potential implications for future PF treatments.

Fostering extensive collaboration and academic exchange among countries can significantly enhance the progress of relevant research endeavors. Through bibliometric analysis, we identified close collaborations among the top four countries (United States, China, Japan, and Germany), indicating comprehensive cooperation in the field of macrophages and PF. The United States and China emerged as the two countries actively contributing to research on macrophages and PF. Notably, 90% of the top 10 institutions are from the United States, while China has shown significant emphasis on research concerning macrophages and PF, with the strongest publication emergence in recent years. Furthermore, our analysis revealed collaborative efforts among research institutions and their publication patterns. It is noteworthy that the top 30 research institutions all originate from the top four countries, indicating that leading research institutions play a dominant role in elevating a country’s academic research ranking. Collaborations among institutions can facilitate the development of research in the related field of macrophages and PF, guiding researchers in publishing high-quality papers in the future.

In terms of research authors, the highest publication counts from Carter, A. Brent, and Kunkel, Sl both originate from the United States, indicating a pivotal role played by the United States in the field of macrophages and PF research. Author co-occurrence analysis in Figure 3B indicates that there are relatively few academic connections between authors from different countries. To advance macrophage and PF research, authors from different countries and institutions should collaborate more closely. Additionally, the significant increase in publications in the past 3 years by Chinese researcher Wang, Jing suggests a heightened focus by this author’s team on the correlation between macrophages and PF during this period.

In terms of research journals, the analysis reveals that a majority of studies on macrophages and PF are published in journals with high international impact, with the American Journal of Respiratory Cell and Molecular Biology, American Journal of Respiratory and Critical Care Medicine, and American Journal of Physiology-Lung Cellular and Molecular Physiology being the most prolific publishers. Furthermore, based on co-citation analysis of journals, research publications and citations related to macrophages and PF are predominantly concentrated in the fields of molecular biology, immunology, and genetics. Future interdisciplinary research can delve deeper into these areas. Particularly noteworthy are the top three cited journals with strong citation burst values: SCI REP-UK, FRONT IMMUNO, and INT J MOL SCI. Their significant increase in citation frequency in the past 3 years indicates growing recognition and suggests that researchers may prioritize these journals for publishing high-quality research papers in the future.

Research on the relationship between macrophages and PF is primarily concentrated in the fields of molecular biology and medicine, with a notable emphasis on the subfield of zoology. This suggests that studies related to macrophages and PF are more oriented toward experimental levels. In the future, exploring the correlation between macrophages and PF through experimental approaches could contribute to better clinical drug applications and disease treatments.

The article titled “Monocyte-derived alveolar macrophages drive lung fibrosis and persist in the lung over the life span” has been cited the most frequently. This study reveals significant heterogeneity in the function of alveolar macrophages during the PF process, holding crucial implications for the design of targeted treatments for PF (14).

In our study, we depicted a co-occurrence network of keywords based on the titles/abstracts of all included publications. Figure 5A illustrates five major research trends categorized into five classes: PF research (red), related diseases (yellow), pathogenesis research (green), diagnosis (blue), and pathology (purple). These results not only align with the anticipated hotspots in macrophage research within the PF domain but also offer insights into potential future research directions: PF research: keywords such as “PF,” “alveolar macrophages,” “bleomycin,” and “cytokines” have been identified as crucial research hotspots. PF is an irreversible progressive lung disease with a poorly understood etiology. Alveolar macrophages, the most common immune cell type in the lungs, play a central coordinating role in tissue response to injury and can induce damage and fibrosis, crucial in the pathogenesis of fibrotic diseases (15). Bleomycin, a complex glycopeptide family with anti-tumor activity, can induce lung inflammation and fibrosis (16). The bleomycin mouse model is widely used to study PF pathogenesis (17). Macrophages release pro-inflammatory cytokines and chemokines during PF, participating in the suppression of inflammation and fibrosis. Research on related lung diseases: “Lung inflammation,” “interstitial pneumonia,” “asthma,” and “chronic obstructive pulmonary disease” are lung diseases related to macrophages and PF. Pathogenesis research: despite the involvement of macrophages in the release of various pro-inflammatory factors during PF, their underlying mechanisms remain incompletely understood. Keywords such as “fibrosis,” “inflammation,” “fibroblasts,” “collagen,” and “macrophage polarization” are currently key focus areas. Diagnostic research: “Bronchoalveolar lavage,” “biomarkers,” and “immunohistochemistry” are research hotspots in the diagnosis of macrophages and PF-related conditions. Pathology research: keywords like “macrophages,” “fibroblasts,” and “alveolar epithelial cells” are hot topics in pathology research related to this study. Recent emerging keywords include “M2 macrophages,” “epithelial-mesenchymal transition,” and “macrophage activation syndrome,” representing current research directions and interests. These findings provide a comprehensive overview of the current state, trends, and hotspots in macrophage-related research within the PF field, offering valuable guidance for future investigations.

TGFβ1, TNF, and CXCL8 are genes with the highest clustering intensity. TGFβ1 (transforming growth factor beta 1): myofibroblasts, characterized by the presence of alpha-smooth muscle actin (α-SMA) and extracellular matrix protein deposition, are essential participants in the PF process. TGFβ1, as a critical pro-fibrotic growth factor, regulates fibroblast proliferation and their transformation into myofibroblasts (18). TGFβ1 plays a key role in promoting the PF process through various signaling pathways, including Smad, MAPK, and ERK pathways, providing new targets for researchers studying novel drugs (19). TNF (tumor necrosis factor): PF involves two major developmental stages: inflammation and fibrosis. Inflammatory cytokines released by macrophages, such as tumor necrosis factor-α (TNF-α), are considered central mediators during the inflammatory phase (20). TNF-α is crucial in inducing epithelial-mesenchymal transition (EMT), a process involved in PF (21). CXCL8 (interleukin-8): changes in the lung’s extracellular matrix (ECM) during PF, such as elastin loss and increased collagen, result in abnormal composition. This affects lung fibroblast proliferation, migration, and differentiation. The pro-inflammatory cytokine CXCL8 responds to aging and promotes the differentiation of ECM-derived cells into myofibroblast phenotypes, potentially contributing to the progression of fibrosis (22).

The GO functional enrichment analysis suggests that these key targets primarily function at the outer side of the plasma membrane, extracellular matrix, vesicle lumen, cytoplasmic vesicle lumen, secretory granule lumen, and membrane raft. They are involved in biological processes such as signaling pathways mediated by cytokines, positive regulation of cytokine production, leukocyte migration, regulation of cell-cell adhesion, positive regulation of the MAPK cascade, and monocyte differentiation. Additionally, they play a role in molecular functions including receptor ligand activity, cytokine receptor binding, cytokine activity, DNA binding transcription factor binding, and G protein-coupled receptor binding.

Kyoto Encyclopedia of Genes and Genomes pathway enrichment analysis reveals cytokine–cytokine receptor interaction, PI3K-Akt signaling pathway, Lipid and atherosclerosis, Kaposi sarcoma-associated herpesvirus infection, and Chemokine signaling pathway as the major pathways. These pathways, such as cytokine–cytokine receptor interaction and PI3K-Akt signaling pathway, are speculated to be key pathways for treating macrophage-related PF. The cytokine–cytokine receptor interaction pathway has the most connected targets, suggesting its close association with the macrophage-related PF process. Cytokine–cytokine receptor interaction pathway in hIGFBP5 pFBs is enriched by differential expression of several chemokines and chemokine receptors implicated in fibrosis development (23). The PI3K/Akt pathway is one of the crucial signaling pathways in the development of PF. Existing evidence suggests that the interaction between PI3K/Akt and transforming growth factor-β (TGF-β) promotes lung fibrosis formation. In multiple stages of PF pathogenesis, the PI3K/Akt pathway interacts with TGF, VEGF, WNT, FAK, mTOR, Jun N-terminal kinase, CTGF, Hedgehog, and Notch pathways (24). As well as lipid and atherosclerosis, Kaposi sarcoma-associated herpesvirus infection, and Chemokine signaling pathway, macrophages and PF also participate in atherosclerosis, viral infection, and chemotaxis. Exploring these pathways may provide guidance for finding drug targets in the treatment of PF. Through the analysis of the correlation between core genes and signaling pathways, it was observed that high-frequency genes such as TGFβ1 and TNF, relevant to macrophages and PF, are primarily associated with pathways such as cytokine–cytokine receptor interaction, AGE-RAGE signaling pathway in diabetic complications, and Lipid and atherosclerosis. Exploring the correlation between core genes and signaling pathways may provide insights into potential therapeutic strategies for the treatment of PF.

In the PPI network, the top five proteins are IL-6, TNF, IL-1B, IL-10, and IFNG, indicating a close relationship with macrophages and PF. Macrophages secrete pro-inflammatory cytokines such as IL-6, IL-1B, and IL-10, promoting the elimination of pathogens and inducing inflammation. The anti-inflammatory cytokines also stimulate collagen and fibroblast production, which are crucial for tissue repair, but can increase the expression of fibrotic factors, resulting in PF (25–27). TNF: lung macrophages exhibit different functional phenotypes under various stimuli and signals, typically either the classical M1 macrophages or the alternatively activated M2 macrophages, both of which play crucial roles in the pathogenesis of PF. Macrophages can be activated into their M1 phenotype, producing the pro-inflammatory cytokine TNF-α. TNF-α can promote aerobic glycolysis and lactate production in lung fibroblasts, thereby facilitating the development of PF (28). Interferon-γ (IFN-γ) and IL-12 have the ability to reduce collagen accumulation and PF. IFN-γ exhibits anti-fibrotic, anti-infective, anti-proliferative, and immune-modulatory properties. It can activate Janus kinase (JAK) 1/signal transducer and activator of transcription (STAT) 1-regulated Smad7 induction, hindering BLM-induced PF (29).

In our analysis of the past 30 years in the field of macrophages and PF, bibliometrics provided a descriptive and quantitative overview. The insights gained from this analysis are crucial for predicting future trends and potential impacts related to PF associated with macrophages. However, this study has certain limitations. For instance, it only included literature published in the last 30 years, searched a single database (WoSCC), and focused solely on English-language publications. Additionally, bibliometric algorithms contained complex black-box models that lacked transparency and interpretability, and could not exclude biases from self-citation and co-authorship citations. These limitations may introduce potential biases.

## Conclusion

In summary, this study constitutes the inaugural scientific and comprehensive bibliometric analysis of the contemporary status and trends in research pertaining to macrophages and PF over the preceding three decades. The investigation methodically encapsulates global publication patterns, delineates research regions, identifies institutions, pertinent authors, and journals germane to the field. Employing methodologies such as hotspot analysis, co-citation analysis, and keyword clustering, the study provides directional insights into major research trajectories within the field, encompassing thematic areas such as “PF research,” “related pulmonary diseases,” “pathogenic mechanisms,” “disease diagnosis,” and “pathological studies.”

Additionally, through GO and KEGG pathway enrichment analysis, coupled with associated disease clustering, the study imparts knowledge to researchers concerning pivotal genes and pathways in the domain. In conclusion, our research findings are poised to steer forthcoming experimental and clinical inquiries into the regulation of macrophages for treating PF. The elucidation of mechanisms governing the interplay between macrophages and PF, along with subsequent molecular biology investigations and mechanistic explorations, holds promise for advancing clinical interventions for PF.

## Data availability statement

The original contributions presented in this study are included in this article/Supplementary material, further inquiries can be directed to the corresponding authors.

## Author contributions

YM: Data curation, Formal analysis, Funding acquisition, Software, Visualization, Writing – original draft, Writing – review & editing. LW: Writing – original draft. CX: Data curation, Writing – review & editing. WH: Data curation, Writing – review & editing. ZY: Formal analysis, Writing – review & editing. XP: Formal analysis, Writing – review & editing. LS: Formal analysis, Writing – review & editing. JZ: Formal analysis, Writing – review & editing. GW: Data curation, Formal analysis, Writing – review & editing. TZ: Data curation, Formal analysis, Writing – original draft, Writing – review & editing.

## Footnotes

1
https://www.citexs.com

2
https://www.graphica.app/

3
http://string-db.org
